# Systematic review and meta-analysis of the diagnostic value of optokinetic after-nystagmus in vestibular disorders

**DOI:** 10.3389/fneur.2024.1367735

**Published:** 2024-02-07

**Authors:** Marie Reynders, Lynn Van der Sypt, Jelte Bos, Wilfried Cools, Vedat Topsakal

**Affiliations:** ^1^Department of Otorhinolaryngology and Head and Neck Surgery, Vrije Universiteit Brussel, Hospital UZ Brussel, Brussels Health Campus, Brussels, Belgium; ^2^Human Performance, Netherlands Organization for Applied Scientific Research (TNO), Soesterberg, Netherlands; ^3^Department of Human Movement Sciences, Vrije Universiteit Amsterdam, Amsterdam, Netherlands; ^4^Interfaculty Center for Data Processing & Statistics, Vrije Universiteit Brussel (VUB), Brussels, Belgium

**Keywords:** Vertigo, nystagmus analysis, optokinetic (OKN) system, optokinetic after-nystagmus, vestibular neuritis, bilateral vestibular areflexia

## Abstract

**Introduction:**

To date, no systematic review or meta-analysis has critically evaluated the relevance of using optokinetic after-nystagmus (OKAN) in diagnosis of vestibular disorders. To assess the role of OKAN in diagnosis of vestibular disorders, the OKAN time constant (TC) between patients with vestibular disorders and healthy participants will be compared.

**Methods:**

Automated search strategies were carried out in the Embase, Medline PubMed, Web of Science, and Scopus databases from inception to December 2023. The following inclusion criteria were applied: (1) evaluation of OKAN in individuals with vestibular disorders, (2) clinical trials, and (3) inclusion of healthy individuals as the control group. Exclusion criteria were: (1) animal studies, (2) non-clinical trial study designs, (3) assessment of non-vestibular disorders, (4) no examination of OKAN TC, (5) only examination of healthy participants, (6) studies published in a language other than English, (7) no healthy participants as control group, (8) case reports, and (9) only abstract available. The random-effects model was used to pool the data. The Joanna Briggs Institute (JBI) Critical Appraisal Tools was used to assess the risk of bias. The quality assessment was performed with the aid of the Quality Assessment Tool for Observational Cohort and Cross-Sectional Studies, provided by NHLBI. The PRISMA guidelines were used as reporting guidelines. The main outcome of this study was the between-group mean difference (MDbetween) in OKAN TC and its 95% confidence interval between patients with vestibular disorders and healthy participants.

**Results:**

Seven out of 244 screened articles were included that studied 289 participants. The overall mean difference (MD = −7.08) with a 95% CI of [−10.18; −3.97] was significant (*p* = 0.014). The heterogeneity was significant (*p* = 0.02). Quality assessment was generally good (76%). The risk of bias was low in five studies and moderate in two studies.

**Conclusion:**

The results demonstrate that OKAN TC is significantly shorter in patients with vestibular disorders compared to healthy controls. This finding is important for future research, particularly with the emergence of novel clinical tools and diagnostic syndromes.

**Systematic Review:**

https://www.crd.york.ac.uk/prospero/display_record.php?RecordID=442695.

## 1 Introduction

Optokinetic nystagmus (OKN) is a repetitive eye movement characterized by an alternating slow and fast phase evoked by a visual stimulus on the retina. A commonly used method to induce OKN is by using a full-field visual pattern rotating around a vertical or horizontal axis aligned with the subject’s head. Frequently used visual patterns in research consist of evenly spaced vertical black and white bars displayed on a drum. The OKN, the smooth pursuit (SP) and the vestibulo-ocular reflex (VOR) are responsible for achieving eye stabilization on the Earth-fixed visual scene (1). The VOR enhances visual stability by coordinating eye movements with head movements to maintain a stable gaze during head rotations (2). The SP is an eye movement in which the eyes track a single moving visual stimulus. Since the SP and OKN are connected, the optokinetic system is best studied through the optokinetic after-nystagmus (OKAN) (3). First described by Ohm in 1921 (4), this neural phenomenon can be recognized as a gradual decrease of the nystagmus after visual stimulation is terminated abruptly. The decrease typically follows an inverse exponential decay characterized by a single time constant (TC). The responsible mechanism for the production of OKAN is thought to be the velocity storage mechanism (VSM), a neural network responsible for multisensory integration (5). The VSM charges when the optokinetic stimulus is observed by the participants. When the visual stimulation is abruptly removed, the VSM slowly discharges and the OKAN occurs as a neurophysiological effect. The general characteristics of OKAN were initially investigated in healthy rhesus monkeys, and later on, the effects of labyrinthectomy in rhesus monkeys on OKAN were explored (5, 6). They found that OKAN TC significantly decreased after unilateral labyrinthectomy. The OKAN TC toward the lesioned side was significantly shorter compared to the OKAN TC toward the healthy side. Moreover, the OKAN TC completely disappeared in bilateral vestibulopathy (7).

The OKAN findings in animals seemed promising for diagnosing vestibular disorders, raising the question as to whether these findings could be applied to humans. Although Cohen et al. (5) found the maximum slow phase eye velocity to be 2–3 times higher in monkeys compared to humans, and Fletcher et al. (8) found the amplitude of the OKAN to be much smaller in humans, these findings did not stop researchers from exploring further the diagnostic possibilities of OKAN in humans. In 1976, Zee et al. (9) examined OKAN in humans to study a cohort of patients suffering from bilateral vestibular loss after meningitis. The findings demonstrated that patients with labyrinthine loss were incapable of generating adequate OKAN velocities when compared to normal patients. The aim of this article is to evaluate the clinical relevance of an abnormal OKAN in medical practice. The systematic overview will examine the available literature on OKAN TC in human vestibular disorders. The meta-analysis aims to assess the between-group mean difference in OKAN TC between individuals with and without vestibular disorders.

## 2 Methods

### 2.1 Protocol review design

This review protocol was registered in the PROSPERO database (CRD42023442695). Two investigators (M.R. and L.V.D.S.) independently performed the same automated search strategy. If there was a disagreement between M.R and L.V.D.S on study inclusion, the judgment of a senior investigator (V.T.) was applied. This systematic review was conducted according to the PRISMA 2020 guidelines (10).

### 2.2 Search strategy

The database search was conducted from inception (1965) to December 2023 in the following databases: Embase, Medline PubMed, Web of Science, and Scopus. The search strategy consisted of the combination of the following keywords: ‘optokinetic after(−)nystagmus,’ ‘vestibular disorders,’ ‘vestibular diagnosis.’ Medical Subject Headings (MeSH) and the main keywords found in related articles were used to determine the keywords for this study. Table 1 displays the search strategy used in different databases.

**Table 1:** Data source and search strategy.

Database	Search strategy
Embase	Title: “optokinetic afternystagmus’ OR ‘optokinetic after-nystagmus” AND “vestibular disorders’ OR ‘vestibular diagnosis”
Medline PubMed	(((optokinetic after-nystagmus) AND (vestibular disorders)) OR ((optokinetic after-nystagmus) AND (vestibular diagnosis))) OR (((optokinetic afternystagmus) AND (vestibular diagnose)) OR ((optokinetic afternystagmus) AND (vestibular disorder)))
Web of Science	(ALL = (optokinetic after-nystagmus)) AND ALL = (vestibular diagnosis) OR (ALL = (optokinetic after-nystagmus)) AND ALL = (vestibular disorders) OR (ALL = (optokinetic afternystagmus)) AND ALL = (vestibular diagnosis) OR (ALL = (optokinetic afternystagmus)) AND ALL = (vestibular disorders)
Scopus	(TITLE-ABS-KEY (optokinetic AND afternystagmus) AND TITLE-ABS-KEY (vestibular AND disorders)) OR (TITLE-ABS-KEY (vestibular AND disorders)) AND (optokinetic AND after-nystagmus) OR (TITLE-ABS-KEY (vestibular AND diagnosis)) AND (optokinetic AND after-nystagmus)

### 2.3 Inclusion and exclusion criteria

Two independent reviewers screened the titles, abstracts, and content of the studies from the selected databases. The inclusion criteria are: (1) evaluation of OKAN in individuals with vestibular disorders, (2) clinical trials, and (3) inclusion of healthy individuals as the control group. The exclusion criteria consist of: (1) animal studies, (2) non-clinical trial study designs, (3) examination of non-vestibular disorders, (4) no examination of OKAN TC, (5) only healthy participants included, (6) studies published in a language other than English, (7) no healthy participants in control group, (8) case reports, and (9) only abstract available.

### 2.4 Data extraction

From the included articles, one researcher extracted the title, authors, year of publication, and sample size (n). The following data on the experiments were collected: the optokinetic stimuli velocity (°/s), the optokinetic stimuli duration (s), TC (s) and standard deviation (SD) for normal participants and vestibular patients. Information regarding the type of studied vestibular disorder was inventoried. In case the OKAN TC was not given explicitly, the TC was generated from the given data by calculating the average OKAN TC per patient and subsequently, the overall average.

### 2.5 Risk of bias and quality assessment

Each included clinical trial was assessed for their risk of bias (RoB). The Joanna Briggs Institute (JBI) Critical Appraisal Tools for use in JBI Critical Appraisal Checklist for Analytical Cross-Sectional Studies was used to assess the RoB (11). The outcomes were independently RoB assessed by two investigators and disagreements were settled by the senior author. An overall low RoB was found when positive answers were above 70%; moderate RoB was considered when positive answers were between 50 and 69%; high RoB was determined when the positive answers were ≤ 49%, recoding to the guidelines. The quality assessment was performed with the aid of the Quality Assessment Tool for Observational Cohort and Cross-Sectional Studies, provided by NHLBI (12). This questionnaire consists of critical questions to determine the quality of the included studies. The questionnaire addresses questions regarding the research question (Q1), the study population (Q2-3), the groups recruited from the same population and uniform eligibility criteria (Q4), sample size justification (Q5), exposure assessed prior to outcome measurement (Q6), sufficient timeframe to see an effect (Q7), different levels of the exposure of interest (Q8), exposure measures and assessment (Q9), repeated exposure assessment (Q10), outcome measures (Q11), blinding of outcome assessors (Q12), follow up rate (Q13) and statistical analyses (Q14). The outcomes were independently assessed by two investigators and disagreements were settled by the senior author.

### 2.6 Statistical analyses

The statistical meta-analysis of this study was conducted using the R Studio software^©^ (2023.03.0 Posit Software). The guidelines provided by Harrer et al. (13) were followed to perform the meta-analyses and obtain the forest plot. The forest plot includes the between-group mean difference (MD_between_), based on the mean OKAN TC from both the patient group and the control group, along with the respective SD and sample sizes from each study. This MD_between_ represents the difference in means between the OKAN TC of the patients versus the controls. The heterogeneity was assessed using the Higgins and Thompson’s *I*^2^ (14). The confidence interval (CI), the weight per study, and the prediction interval were determined.

### 2.7 Sensitivity analysis

In anticipation of potential variability in the OKAN TC, a sensitivity analysis was pre-planned, given the ongoing debate surrounding the preferred model choice. No *a priori* subgroup analysis was planned.

## 3 Results

### 3.1 Study selection

**Figure 1:**
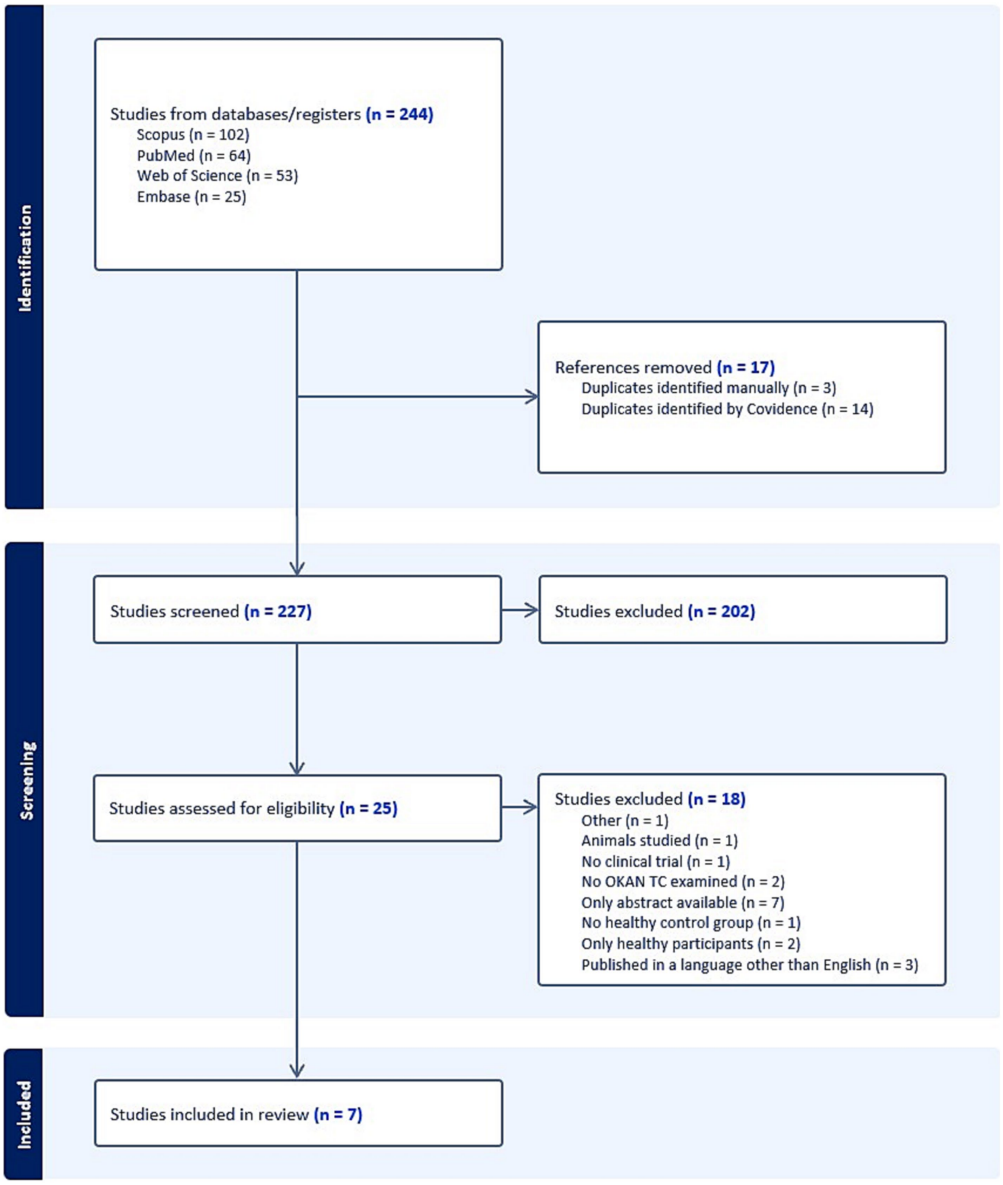
PRISMA flowchart diagram displaying search results. BMJ (Open access) (10).

Figure 1 shows the results of the search strategy delivering 244 articles. After removal of duplicates and after the studies were screened by abstract and title, 25 studies were screened for full-text eligibility. Eventually, seven articles were included for meta-analysis. For those articles that met the inclusion criteria but for which only the abstract was available, we contacted the authors. As such, we received two articles by Brantberg et al. (15, 16) through the Karolinska Institute, which were included in the study. The studies conducted by Hain et al. in 1994 included 90% of the same patients as their study conducted in 1992, information we received through personal communication with Prof. Dr. Hain. For this reason, we decided not to include the article from 1994 (exclusion criteria: “other”).

### 3.2 Study characteristics

The seven included studies involved a total of 289 participants. To elicit the horizontal optokinetic stimulus, all studies used a whole-field optokinetic drum displaying alternating white and black stripes. The optokinetic stimulus duration and velocity, and the vestibular conditions are outlined in Table 2. For all articles, exclusively OKAN I (the first phase of OKAN) was used to calculate the TC. In the study of Jell (18), it is noteworthy that the OKAN TC is relatively high for the vestibular group (95.3 s) and the control group (48.8 s), compared to the other studies. However, two patients within this group exhibited an exceptionally high OKAN TC on one side, which appeared inconsistent with the values observed in other patients. The articles did not provide any explanation for these outliers. As seen in Table 2, the last three studies utilized a stimulus duration of 87 s, while the first three studies used a stimulus duration of 60 s. The OKAN TC was significantly higher in the studies that used a 60-s versus 87-s stimulus duration (*p* < 0.05).

**Table 2:** Overview of the articles included.

*Study nr.*	Author	Sample size (n,n,n)	OKAN		Optokinetic stimulation		Condition
			*TC (s) + SD of control*	*TC (s) + SD of patients*	*Velocity (°/s)*	*Duration (s)*	
1	Zasorin et al. (1983) (17)	^a^T: 35 P: 15 C: 20	23.7 ± 23.1	7.8 ± 11.4	60	60	Bilateral hyporeflexia
2	Jell et al. (1988) (18)	T: 25 P: 11 C: 14	48.8 ± 26.7	95.3 ± 166.12	40	60	Mixed
3	Brantberg and Magnusson (1990) (16)	T: 44 P: 20 C: 24	24.7 ± 16.4	13.5 ± 11.3	90	60	Unilateral acoustic neurinoma
4	Brantberg and Magnusson (1991) (15)	T: 64 P: 14 C: 50	27.6 ± 21.6	21.1 ± 15.4	90	60	Unilateral vestibular neuritis
5	Hain and Zee (1991) (19)	T: 38 P: 8 C: 30	12 ± 7.4	3.7 ± 1.0	60	87	Aminoglycoside induced bilateral vestibulopathy
6	Hain et al. (1992) (20)	T: 43 P: 13 C: 30	11.3 ± 3.2	7.2 ± 1.8	60	87	Unilateral surgical section of the vestibular nerve (due to acoustic neurinoma surgery)
7	Dellepiane et al. (2006) (21)	T: 40 P: 20 C: 20	9 ± 6.2	1.7 ± 1.9	30	87	Bilateral hyporeflexia

^a^T, total number of subjects; P, number of patients; C, number of controls. TC, time constant.

### 3.3 Risk of bias and quality assessment

Table 3 provides the specific assessment of the RoB for each individual study. In the studies conducted by Hain et al. (19, 20) and Dellepiane et al. (21), the optokinetic stimulus duration was extended to 87 s. This deviated from the recommended duration of 60 s in light of our own observations and findings from previous studies (22). Since this is an important confounder for OKAN, the answer to the confounders’ question yielded a negative result (Q5). Furthermore, none of the studies implemented strategies to deal with the effects of other confounding factors, which could have been accounted for through a form of multivariate regression analysis (Q6). In the studies of Jell et al. (18) and Dellepiane et al. (21), not all the patients received a specified diagnosis (Q4). The inclusion criteria in these studies were also somewhat ambiguous (Q1). After the JBI criteria were applied, five studies demonstrated a low Risk of Bias (RoB), while two raised some concerns regarding RoB. For the quality assessment, all studies scored NO for question 2 because the group of participants was not selected based on demographics or location. Question 5 was also marked NO for all studies because no sample size was calculated in advance. Question 6 was marked NO for all studies because the study design was cross-sectional. Blinding was not possible, therefore question 12 was marked as NA (not applicable). Taking all this into account, the overall quality of the included studies has a good (76%, 10/13 with one point not applicable) rating for methodological quality.

**Table 3:** Risk of bias assessed by the Joanna Briggs Institute (JBI) Critical Appraisal Tools.

Authors	Q1	Q2	Q3	Q4	Q5	Q6	Q7	Q8	% Yes	Risk
Jell et al. (18)	−	√	√	−	√	−	√	√	62.5%	Moderate
Brantberg et al. ‘90 (16)	√	√	√	√	√	−	√	√	87.5%	Low
Brantberg et al. ‘91 (15)	√	√	√	√	√	−	√	√	87.5%	Low
Hain et al. ‘91 (19)	√	√	√	√	−	−	√	√	75%	Low
Hain et al. ‘92 (20)	√	√	√	√	−	−	√	√	75%	Low
Dellepiane et al. (21)	−	√	√	−	−	−	√	√	50%	Moderate
Zasorin (17)	√	√	√	√	√	−	√	√	87.5%	Low

√ Yes; − No;

Q1. Were the criteria for inclusion in the sample clearly defined?

Q2. Were the study subjects and the setting described in detail?

Q3. Was the exposure measured in a valid and reliable way?

Q4. Were objective, standard criteria used for measurement of the condition?

Q5. Were confounding factors identified?

Q6. Were strategies to deal with confounding factors stated?

Q7. Were the outcomes measured in a valid and reliable way?

Q8. Was appropriate statistical analysis used?

### 3.4 Forest plot

**Figure 2:**
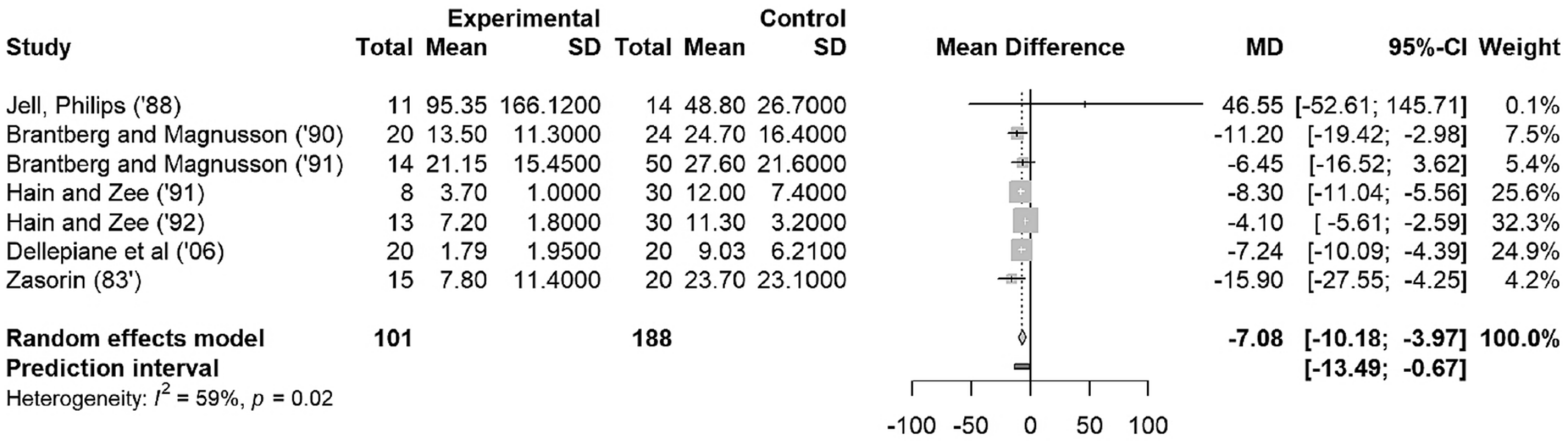
Forest Plot on OKAN TC. The squares represent the mean difference for each study. The size of the squares are proportional to the weight per study. Error bars indicate the 95% confidence intervals of mean difference. The diamond represents the overall mean difference.

In the meta-analysis no distinction was made between uni- or bilateral vestibulopathy, but rather the overall effect size between vestibular patients and controls was calculated, because not all studies made a distinction between uni- and bilateral vestibulopathies. Figure 2 shows a forest plot to provide an accurate representation of the effect sizes. The studies of Hain et al. (16), carry the highest weight of all included studies. The random-effects model resulted in an effect size of −7.08 with a CI 95% of [−10.18; −3.97]. The *p* value for this random-effects model was 0.0014 (not shown in plot), indicating a significant difference in OKAN TC between patients and healthy participants. The heterogeneity is expressed through *I*^2^ (59.1%) and the *p* value (0.02) indicating the heterogeneity is moderate and significant, respectively, (Figure 2) (12, 14). The prediction interval (−13.49; −0.67) estimates the interval of effect size for a new study.

### 3.5 Sensitivity analysis

According to the Cochrane Handbook for Systematic Reviews of Interventations, it is recommended that a random-effects model should be used in the presence of heterogeneity (23). However, if we applied the fixed-effects model, the MD was found to be −5.68 with 95% CI of [−6.85; −4.51]. The mean difference between fixed-effects model and random effects-model is not large (−5.68 vs. −7.08), so the influence of the design decision is negligible.

## 4 Discussion

To our knowledge, this systematic review provides the most up-to-date and comprehensive evidence of the use of OKAN in vestibular disorders, summarizing estimates from seven original studies. The OKAN TC in patients with peripheral unilateral vestibulopathy was found to be asymmetrical compared to the symmetrical OKAN TC in healthy patients according to both Brantberg and Magnusson (15, 16), and Hain et al. (20). Additionally, the former authors found that the asymmetry in OKAN TC seemed to normalize in patients that recovered from vestibular neuritis. This observation indicates that OKAN TC can be a helpful measure in tracking recovery or improvements in individuals with vestibular neuritis. In bilateral vestibulopathy, Hain et al. (19), Zasorin et al. (17), and Dellepiane et al. (21) found that OKAN TC was symmetrically significantly reduced compared to the OKAN TC in healthy subjects. Lastly, Jell et al. (18) showed that despite caloric tests had returned to normal values, simultaneous measurements of OKAN still exhibited persistent asymmetry in patients suffering from multiple vestibular pathologies. These findings suggest that OKAN measurements might provide additional insights into vestibular function beyond what is captured by traditional caloric tests.

The overall analysis showed a significant difference in the OKAN TC between people with vestibular deficits and those without. In addition, the prediction interval indicates, with 95% certainty, the OKAN TC of patients will be different from that of controls in future studies. This means the observed difference is quite reliable and likely to be consistent in similar studies down the line.

In summary, OKAN TC is found to be asymmetric in unilateral vestibulopathies, reduced bilaterally in bilateral vestibulopathies, symmetric in healthy patients and appears to lose asymmetry in recovering pathologies. Therefore, OKAN TC could be a tool in monitoring the recovery process of patients, which is an added value for several reasons. This can help healthcare professionals to track changes in functionality and give patients a tool that records their recovery progress. However, further research is warranted to integrate OKAN into clinical settings effectively.

In the past, the use of OKAN has been studied in well-known vestibular pathologies such as vestibular neuritis. With the emergence of new technologies such as cVEMP and vHIT, the use of OKAN has become largely redundant. Nevertheless, while these tests focus mainly on the SCC and the otoliths, OKAN measurements can offer crucial insights into the visuo-vestibular pathway. Recent research suggests that visual dependency, dizziness triggered by seeing movements, moving images or busy patterns, can be objectively assessed through OKAN (24). It is hypothesized that the velocity storage mechanism (VSM), the central system responsible for multisensory integration, is impacted in individuals with visual dependence. Therefore, the VSM discharges more slowly after the removal of visual stimulation, leading to an extended duration of OKAN in individuals with visual dependence (25–28). This prompts the question as to whether OKAN could potentially serve in more recently discovered diagnoses like persistent postural-perceptual dizziness (PPPD) and vestibular migraine—conditions characterized by visual dependency for which objective diagnostic tests are currently lacking (29). Further research is necessary to define the diagnostic use of OKAN TC in PPPD.

Importantly, the meta-analysis uncovered insights not apparent in individual studies. When analyzing our data, the 60-s stimulus duration exhibited a significantly higher average OKAN TC compared to the studies using an 87-s stimulus duration. Combining prior research with our own, a 60-s stimulus duration seems to be the preferred choice for determining OKAN TC (30). However, important questions remain unclarified. For instance, the ideal stimulus velocity that would gain the most accurate outcome is unknown. Furthermore, we are unsure about how many seconds after the stimulus has terminated, we must measure OKAN to not let the OKN interfere with the SP. Unfortunately, too little research has been done to provide sufficiently informed answers to those questions. Nevertheless, these are aspects to consider while executing future research on OKAN.

It is noteworthy that only seven articles were included in this review. This is likely due to the strict inclusion criteria, e.g., only English-language articles were included. This could possibly contribute to the research finding of only a small number of publications on a topic that has been extensively researched in the past. Furthermore, several articles included in this meta-analysis are relatively outdated. However, the quality assessment and risk of bias demonstrate that the studies are generally well-constructed. In addition, findings from more recent studies do not differ from the oldest ones. Although the results seem promising, the use of OKAN is currently used in study set-up but not in clinical practice. An obvious explanation is because generating OKAN has traditionally posed challenges by the spatial requirements and complex experimental set-ups. For example, OKAN is only elicited by large field stimuli and the darkness after stimulus should be completely dark. However, with ongoing technological advancements, there is potential for improving the feasibility of OKAN measurements in clinical practice. The landscape of medical technology is undergoing a transformative shift toward innovative technology such as Virtual and Augmented Reality, and Artificial Intelligence. These technologies hold the potential to regain interest in the approach of OKAN measurement and its diagnostics use.

## 5 Limitations

The risk of selection bias exists since restrictions during inclusion were applied. For example, articles that were not included in this study examined OKAN findings in central pathologies such as multiple sclerosis and concussion. This decision was made to ensure comprehensible conclusions. Since OKAN is a very specific topic, a limited number of papers were included in the meta-analyses. However, the reduction in OKAN TC is a consistent finding across almost all investigated studies. We conclude that a 60 s stimulus duration is the most ideal choice for gaining the highest mean OKAN TC realizing that cofounders were not included in statistical analysis. Our future research will focus on this item.

## 6 Conclusion

This meta-analysis suggests that OKAN TC is a useful examination for vestibular deficits. The identified preference for a 60-s stimulus duration, in line with previous research, can be a guide for practical application of OKAN assessments in clinical settings. OKAN is not currently used for vestibular function measurement due to its large and complex set-up, but it could be useful in conditions where the visuo-vestibular pathway is impaired.

## Data availability statement

The original contributions presented in the study are included in the article/supplementary material, further inquiries can be directed to the corresponding author.

## Author contributions

MR: Conceptualization, Data curation, Formal analysis, Investigation, Methodology, Project administration, Visualization, Writing – original draft, Writing – review & editing. LS: Conceptualization, Investigation, Methodology, Writing – original draft, Writing – review & editing. JB: Conceptualization, Writing – original draft, Writing – review & editing. WC: Formal analysis, Methodology, Visualization, Writing – original draft, Writing – review & editing. VT: Conceptualization, Writing – original draft, Writing – review & editing, Supervision, Validation.
